# A nomogram for predicting postoperative urosepsis following retrograde intrarenal surgery in upper urinary calculi patients with negative preoperative urine culture

**DOI:** 10.1038/s41598-023-29352-y

**Published:** 2023-02-06

**Authors:** Miaomiao Yang, Yongchao Li, Fang Huang

**Affiliations:** 1 The Second Xiangya Hospital, Central South University, Changsha, 410012 Hunan China; 2 Department of Urology, Xiangya Hospital, Central South University, Changsha, 410008 Hunan China

**Keywords:** Predictive markers, Risk factors, Urology

## Abstract

Retrograde intrarenal surgery (RIRS) is one of the main surgical methods for upper urinary calculi, but severe complications of infection may occur after surgery. This study aimed to establish and validate a preoperative nomogram for predicting postoperative urosepsis following retrograde intrarenal surgery to treat upper urinary calculus in patients with a negative preoperative urine culture. We retrospectively recruited 1767 patients with negative preoperative urine cultures who underwent retrograde intrarenal surgery to treat upper urinary calculi from January 2017 to April 2022. The independent risk factors for urosepsis include a solitary kidney, positive urine nitrite, operative time ≥ 75 min, history of recurrent urinary tract infections, and history of diabetes were identified by univariate analysis and multivariate binary logistic regression analysis, which construct a nomogram. The receiver operating characteristic curve of the nomogram for predicting urosepsis was 0.887 in the training cohort and 0.864 in the validation cohort, respectively. The calibration curve and decision curve analysis demonstrated great consistency and clinical utility of the nomogram. Therefore, the nomogram combining preoperative independent risk factors can predict the probability of a postoperative urosepsis following retrograde intrarenal surgery in patients with a negative preoperative urine culture, which could help urologists take preventive measures in advance after surgery to avoid more serious complications of infection.

## Introduction

The incidence of symptomatic kidney stones requiring treatment has increased recently^1^. Retrograde intrarenal surgery (RIRS), a well-established minimally invasive procedure, has become one of the main surgical methods for treating upper urinary calculi^2,3^. However, this procedure could result in some serious complications^4^, especially postoperative infection, which can develop into life-threatening urosepsis^5^. Therefore, urologists have been concerned about how to avoid the occurrence of postoperative infectious complications. Transmission of pelvic bacteria or toxins into the bloodstream during surgery is currently considered to be the most likely cause of sepsis^6,7^; therefore, positive preoperative urine culture (UC) is an important risk factor for postoperative infection complications following RIRS^8,9^. However, preoperative UC cannot adequately reflect renal pelvis urine and stone, and negative preoperative UC cannot exclude the risk of postoperative infection complications^10–12^. Previous studies reported that an incidence of septic shock (1.09%) was noted in patients with negative preoperative UC^13^, which indicated that patients with negative UC were still at risk of serious infection complications after surgery. It has been reported that calculi and intraoperative UC are better than preoperative UC in predicting the occurrence of postoperative infection complications^10,11,14^. However, infection complications generally occur within 24 h after surgery, and stones and an intraoperative urine culture cannot provide early warning for clinicians to prevent postoperative infectious complications. Considering the potential risk of urosepsis in patients with negative UC and the lack of analysis of risk factors associated with urosepsis in patients with negative UC after RIRS. Thus, this study aimed to develop and validate a nomogram for predicting the likelihood of postoperative urosepsis by evaluating the preoperative variables from patients with negative preoperative UC.

## Materials and methods

### Patients selection

Patients with negative preoperative UC who underwent RIRS from January 2017 to April 2022 were retrospectively recruited. The limited number of patients with specific conditions (such as simultaneous bilateral RIRS, repeated ureteral malformation, and horseshoe kidney) during the study period may have seriously masked the true nature of the study. Therefore, these special cases were excluded from this study. Patients were excluded according to the following exclusion criteria: (1) patients aged < 18 years;(2) patients undergoing bilateral procedures or procedures that were simultaneously combined with other surgeries; and (3) patients with special situations such as pregnancy, duplicate ureteral deformity and horseshoe kidney. A total of 1767 patients were eventually enrolled in this study, of which 1060 patients from January 2017 to March 2020 were used as the training cohort, and 707 patients from April 2020 to April 2022 were used as the validation cohort. A review of the patient's medical records found no evidence of medication use (e.g., malofen, furantoin, sulfapyridine) or disease (e.g., jaundice) within the two weeks before surgery that affected the results of the urine nitrite test. All operations were performed by three senior urologists with over eight years of experience in minimally invasive surgery. All patients received a ureteral access sheath (UAS, F12/14, 45 cm for males, 35 cm for females) and a gravity water bag (height 90–100 cm) infusions (The procedure is detailed in Supplementary information).

All patients underwent a plain film of kidney-ureter-bladder (KUB) and abdominal non-contrast computed tomography (CT) scan preoperatively to evaluate the stone size, position, and hardness (measured in Hounsfield units, HU). Midstream urine culture was routinely performed one week before surgery after determining that the patient had not received antibiotic treatment in the last two weeks. A negative UC was defined as isolated bacteria less than 10^4^ cfu/ml. A single dose of prophylactic antibiotics was administered to all patients 30 min before the anesthetic, and the antibiotics were routinely continued less than 24 h postoperatively. When the patients developed urosepsis, higher levels of antibiotics, such as third-generation cephalosporin or imipenem, were replaced in time. Continuous electrocardiograph monitoring was performed routinely within 24 h after the operation. The study was conducted by the Helsinki Declaration. The Ethics Committee of the Xiangya Hospital of Central South University approved all procedures performed in this study.

### Data collection and outcome assessment

If multiple stages of RIRS were performed, only the first stage was included. Basic clinical information of all patients was collected by retrospective review of electronic medical records. Body Mass Index (BMI) was defined as weight in kilograms divided by height in meters squared (kg/m^2^). According to the characteristics of Chinese people, BMI ≥ 28 kg/m^2^ is defined as obesity^15^. Through the review of anamnesis of the medical records, a history of recurrent urinary tract infections (rUTIs) was defined as patients' self-reported urinary tract infections ≥ 2 times within 6 months or ≥ 3 times within 1 year^16^. The extent of hydronephrosis was assessed according to the Society of Fetal Urology grading system^17^. Stone volume was calculated using the following formula: 0.167 × π × Length_max_ × Width_max_ × Depth_max_, and the burden of multiple stones was calculated as the sum of the volume of all the stones. Postoperative fever was defined as a temperature of > 38 °C. Urosepsis has defined as ≥ 2 criteria of the quick sepsis-related organ failure assessment (qSOFA):(1) respiratory rate of ≥ 22 breaths/min; (2) altered consciousness (Glasgow Coma Scale score of < 13); and (3) systolic blood pressure of ≤ 100 mmHg^18^. In addition, medical images of all patients were independently read by a radiologist and a urologist to measure the calculi burden as determined by CT The comorbidities of patients were evaluated by the age-adjusted Charlson Comorbidity Index (aCCI)^19^.

### Statistical analyses

The chi-square test or Fisher's exact test was used to analyze the proportion of categorical variables; Student's t-test was used to analyze numerical variables with normal distribution, and the Mann–Whitney U test was performed to analyze numeric variables that were not properly distributed, and multivariate binary logistic regression models were performed to determine the independent risk factors for postoperative urosepsis following RIRS. Variables showing a *p*-value < 0.05 in univariable analysis were considered candidates for the stepwise forward multivariable logistic regression model based on maximum likelihood estimation. Multicollinearity was detected before multivariate logistic regression analysis. Based on the regression coefficients of the independent variables, an individualized nomogram prediction model of urosepsis was constructed. Using the area under the receiver operating characteristic curve (AUC-ROC) evaluated the nomogram model's predictive accuracy and cut-off value. The score corresponding to the cut-off value of the ROC curve is the cut-off point of the total score on the nomogram model. The calibration curve analysis with 1000 bootstraps re-sample was used to assess the calibration performance of the nomogram. Decision curve analysis (DCA) further evaluated the clinical validation of the nomogram. *p*-values < 0.05 derived from the two-tailed test were considered statistically significant. All statistical analyses were performed using the Statistical Package for the Social Sciences 22.0 (SPSS for Windows, Chicago, IL, USA) and STATA (version 15.1).

### Ethical statement

The authors are accountable for all aspects of the work, including ensuring that questions related to the accuracy or integrity of any part of the work have been appropriately investigated and resolved. The study was conducted by the Helsinki Declaration (as revised in 2013). All procedures performed in this study were approved by the Ethics Committee of the Xiangya Hospital, Central South University, and the informed consent of all the patients was obtained.

### Consent to participate

Written informed consent to surgical procedures and the publication of clinical data on the condition of anonymity was obtained preoperatively from all included patients.

## Results

### Descriptive analysis of the patient population and determination of the independent predictive factors for urosepsis in the training cohort

A total of 1060 patients with preoperative negative UC who underwent RIRS were enrolled in the training cohort. The characteristics of the patients in the training cohort are shown in Table 1. Twenty-nine (2.7%) patients developed urosepsis following RIRS. The mean age of the no urosepsis group was 51.4 ± 12.5, and 66.7% of the patients were male, including 41(4.0%) patients with a history of rUTIs, 219(21.2%) patients with hypertension, 88(8.5%) patients with diabetes. The mean age of the 29 patients with urosepsis was 47.5 ± 12.8, which included 20(69.0%) males, 4(13.8%) patients with a history of rUTIs, 3(10.3%) patients with hypertension, and 6(20.7%) patients with diabetes. Univariate analysis showed that urosepsis was related to diabetes (*p* = 0.023), a history of rUTIs (*p* = 0.014), solitary kidney stones (*p* < 0.001), a history of urolithiasis surgery (*p* = 0.001), an operative time ≥ 75 min (*p* < 0.001), stone burden (*p* = 0.014), and positive urine nitrite (*p* < 0.001), but not to sex, hypertension, surgeons, obesity, degree of hydronephrosis, stone location, and stone hardness. The onset of urosepsis significantly prolonged the length of postoperative hospital stay (6.6 vs. 2.2 days, *p* < 0.001), as shown in Table 1.

**Table 1:** The clinical characteristics of patients with or without postoperative urosepsis in the training cohort.

Risk factors	No urosepsis (N = 1031)	Urosepsis (N = 29)	*p*-value
Age, years, mean (SD)	51.4(12.5)	47.5(12.8)	0.119
Sex			0.801
Male (%)	688(66.7)	20(69.0)	
Female (%)	343(33.3)	9(31.0)	
aCCI > 3, n (%)	68(6.6)	3(10.3)	0.426
Hypertension, n (%)	219(21.2)	3(10.3)	0.155
Diabetes, n (%)	88(8.5)	6(20.7)	0.023
Surgeon, n (%)			0.599
Surgeon A	310(30.1)	11(37.9)	
Surgeon B	360(34.9)	10(34.5)	
Surgeon C	361(35.0)	8(27.6)	
History of rUTIs, n (%)	41(4.0)	4(13.8)	0.014
History of urolithiasis surgery, n (%)			0.001
None	700(67.9)	12(41.4)	
PCNL	112(10.9)	9(31.0)	
RIRS or URL	156(14.7)	8(27.6)	
ESWL	44(4.3	0(0.0)	
Open operation	19(1.8)	0(0.0)	
Pre-stented, n (%)	117(11.3)	5(17.3)	0.327
Obesity, n (%)	97(9.4)	5(17.2)	0.158
Solitary kidney stone, n (%)	116(11.3)	10(34.5)	< 0.001
Degree of hydronephrosis, n (%)			0.982
None or mild	961(93.2)	27(93.1)	
Moderate or severe	70(6.8)	2(6.9)	
Stone location, n (%)			0.105
Upper segment of ureter	409(39.7)	6(20.7)	
Kidney	433(42.0)	17(58.6)	
Ureteral with kidney	189(18.3)	6(20.7)	
Stone burden, mm^3^, mean (SD)	624.2(421.7)	819.9(449.8)	0.014
Stone hardness (HU), mean (SD)	1004.8(314.0)	889.3(341.6)	0.082
ASA, n (%)			0.133
Class 1 and 2	705(68.4)	16(55.2)	
Class 3 and 4	326(31.6)	13(44.8)	
Preoperative blood WBC count(10^9^/L), mean (SD)	6.2(1.8)	6.8(1.9)	0.096
Preoperative neutrophil granulocyte rate, mean (SD)	59.0(9.2)	58.7(4.6)	0.868
Preoperative hemoglobin, g/L, mean (SD)	132.7(17.8)	126.8(16.1)	0.082
Hct level, mean (SD)	41.1(20.6)	38.2(5.0)	0.457
Serum creatinine, µmol/L, mean (SD)	110.6(97.7)	101.2(25.9)	0.603
Positive urine WBC, n (%)	805(78.1)	27(93.1)	0.064
Positive urine nitrite, n (%)	61(5.9)	18(62.1)	< 0.001
Operative time ≥ 75 min, n (%)	290(28.1)	20(69.0)	< 0.001
Postoperative hospital stay, days (SD)	2.2(0.8)	6.6(3.6)	< 0.001

*aCCI* age-adjusted Charlson Comorbidity Index; *rUTIs* recurrent urinary tract infections; *PCNL* percutaneous nephrolithotomy; *RIRS* retrograde intrarenal surgery; *URL* ureteroscopy lithotripsy; *EWSL* extracorporeal shock wave lithotripsy; *ASA* American Society of Anesthesiologists; *WBC* white blood cells; *Hct* level hematocrit level; *SD* standard deviation.

Subsequently, the variables with *p* < 0.05 in the univariate analysis were included in the stepwise forward multivariable logistic regression model based on maximum likelihood estimation. The risk factors such as a solitary kidney stone, history of rUTIs, positive urine nitrite, history of urolithiasis surgery, operative time, stone burden, and history of diabetes were incorporated into this model. Finally, the results indicated that solitary kidney stones (OR: 4.9, 95%CI: 1.9–12.4, *p* = 0.001), positive urine nitrite (OR: 21.3, 95%CI: 9.1–50.0, *p* < 0.001), a history of rUTIs (OR: 6.0, 95%CI: 1.7–21.6, *p* = 0.006), an operative time ≥ 75 min (OR: 4.5, 95%CI: 1.8–11.4, *p* = 0.001), and history of diabetes (OR: 3.4, 95%CI: 1.1–10.5, *p* = 0.029) were independent risk factors for urosepsis following RIRS (Table 2).

**Table 2:** Multivariate logistic regression analysis of risk factors for urosepsis following RIRS.

Risk factors	OR (95% CI)	*p*-value
Solitary kidney stone, (yes/no)	4.9(1.9–12.4)	0.001
History of rUTIs, (yes/no)	6.0(1.7–21.6)	0.006
Positive urine nitrite, (yes/no)	21.3(9.1–50.0)	< 0.001
Diabetes, (yes/no)	3.4(1.1–10.5)	0.029
Operative time ≥ 75 min, (yes/no)	4.5(1.8–11.4)	0.001

*rUTIs* recurrent urinary tract infections; *OR* odds ratio; *CI* confidence index.

### Development and validation of the nomogram

**Figure 1:**
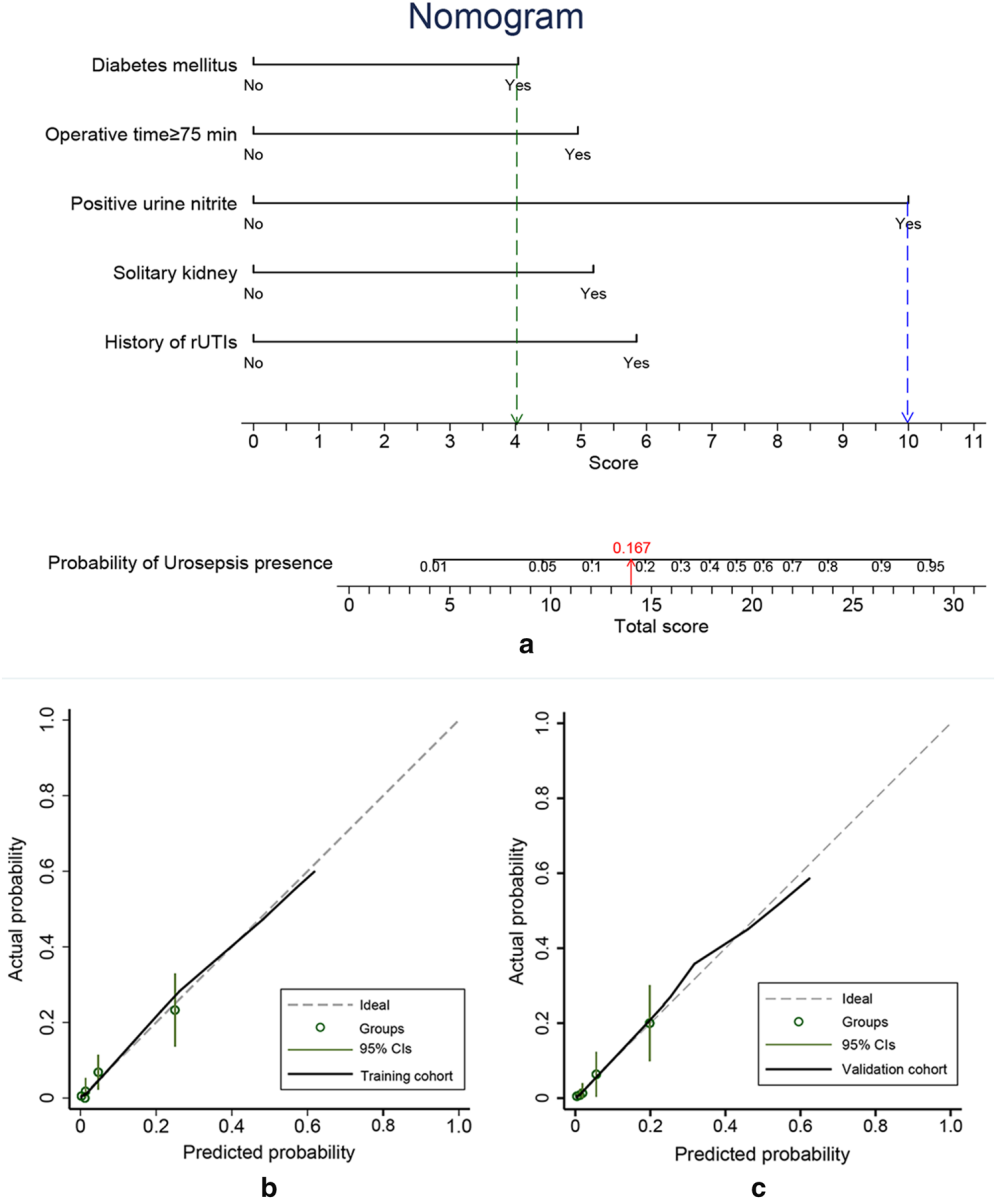
Nomogram and calibration plot. (**a**) Nomogram for predicting the probability of urosepsis in patients after RIRS. To use the nomogram, draw a vertical line to identify the corresponding score of each variable according to their actual status. Then sum up the score of all variables and find the position on the total score axis. The probability of urosepsis can be obtained by drawing a vertical line from the “Total score” axis to the “Probability of Urosepsis presence” axis. For example, if a patient has diabetes mellitus (green line; 4 scores) and positive urine nitrite (blue line; 10 scores) before RIRS, we calculate the total score as 4 + 10 = 14. By drawing a vertical line (red line) from this score on the “Total score” axis to the “Probability of Urosepsis presence” axis, it concludes that the probability of the patients developing urosepsis after RIRS is 16.7%. (**b**) Calibration curve of the nomogram using 1000 bootstrap re-samples in the training cohort. (**c**) Calibration curve of the nomogram using 1000 bootstrap re-samples in the validation cohort. The ideal reference line represents that the predicted likelihood perfectly matches the actual incidence.

Based on these independent risk factors, a nomogram prediction model was established to calculate the cumulative probability of urosepsis in UC-negative patients after RIRS (Fig. 1a). The calibration curve was developed using 1000 bootstrap re-samples in both cohorts and showed great consistency between the risk predicted by the nomogram and observed outcomes (Fig. 1b,c).

**Figure 2:**
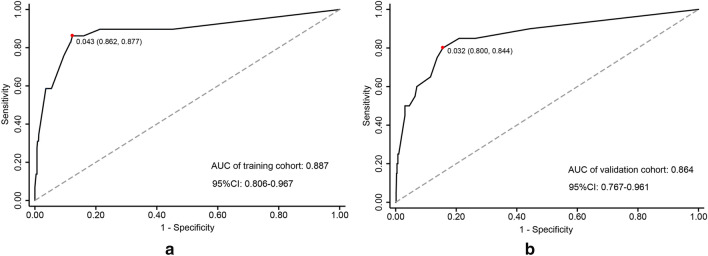
ROC curves of the nomogram for predicting urosepsis. (**a**) ROC curve of the nomogram with an AUC of 0.887 and a cut-off value of 0.043 (sensitivity: 86.2%, specificity: 87.7% ) in the training cohort. (**b**) ROC curve of the nomogram with an AUC of 0.864 and a cut-off value of 0.032 (sensitivity: 80.0%, specificity: 84.4%) in the validation cohort.

The ROC curve was used to evaluate the predictive accuracy of the nomogram, which demonstrated a moderate predictive power in the training cohort (AUC, 0.887; 95% CI, 0.806–0.967) and validation cohort (AUC, 0.864; 95% CI, 0.767–0.961). The optimal cut-off value of the ROC curve in the training and validation cohorts was 0.043 (sensitivity: 86.2%, specificity: 87.7%) (Fig. 2a) and 0.032 (sensitivity: 80.0%, specificity: 84.4%) (Fig. 2b), respectively. Based on the optimal cut-off value of the training cohort, we found that the nomogram's corresponding cut-off value of the total score was 9.2. Patients with scores higher than 9.2 were at high risk for urosepsis after RIRS.

**Figure 3:**
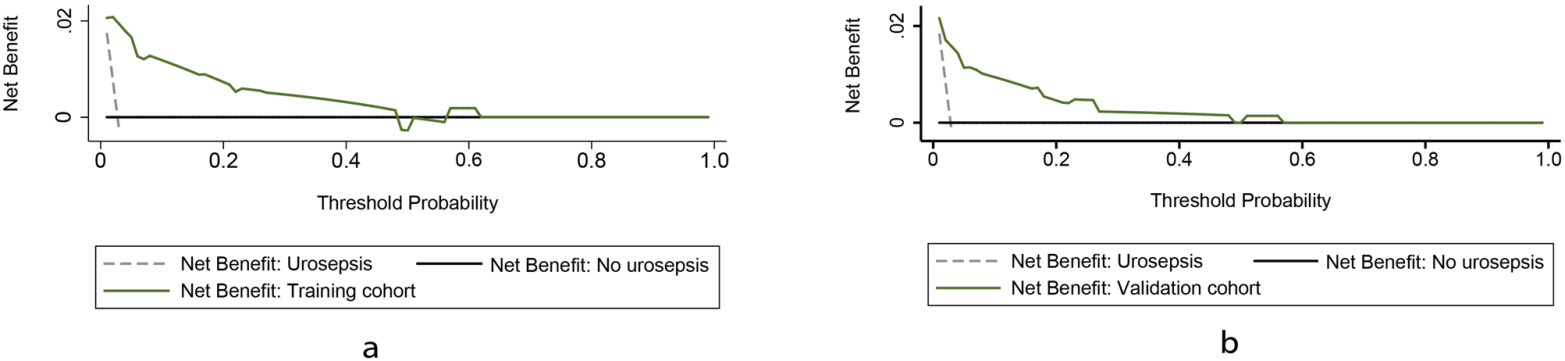
The decision curve of the nomogram plotting the net benefit versus threshold probabilities in the training (**a**) and validation (**b**) cohorts. The black line plots the assumption that all patients have urosepsis, whereas the dotted line assumes none have urosepsis. The green line is the clinical benefit of using the nomogram model, showing benefit in predicting a urosepsis at a threshold probability of the range from about 0 to 0.5.

The decision curve analyses showed that patients would gain a significant positive benefit from the nomogram when the threshold probability for a patient was within the range of 2%–50% in the training and validation cohort (Fig. 3).

## Discussion

It has been reported that the incidence of postoperative RIRS infection is up to 25.0%^4^, and urosepsis can be life-threatening^20^. Therefore, the widespread use of preoperative prophylactic antibiotics is an accepted strategy to prevent perioperative infection^2,21^. However, drug resistance and opportunistic infections may increase with the overuse and misuse of antibiotics^22^. Moreover, the consensus on antibiotic prophylaxis in the treatment of endoscopic calculi remains controversial, especially in patients with preoperative sterile urine^23,24^. To the best of our knowledge, the analysis of risk factors associated with postoperative infectious complications in patients with negative urine cultures after RIRS is still lacking. In the current study, despite the negative preoperative UC, 2.7% of patients developed urosepsis according to qSOFA. These results are consistent with a previous report on RIRS in patients with negative UC whose incidence of infection-related complications ranged from 0 ~ 17%^24^. Therefore, more attention should be given to postoperative infection after RIRS in patients, despite negative preoperative UC.

In this study, univariate analysis and multivariate logistic regression analysis conferred solitary kidney, a history of rUTIs, positive urine nitrite, an operative time ≥ of 75 min, and a history of diabetes were significant risk factors for postoperative urosepsis in patients with negative U.C. Urine nitrite is widely used in the diagnosis of UTIs and serves as an essential risk factor for predicting complications of UTIs after intracavitary surgery^9,25^. Meanwhile, a prolonged operation time leads to a continuous increase in renal pelvis pressure, which increases the chance of bacteria or toxins from renal pelvis urine entering the bloodstream, thus increasing the chance of postoperative infection complications^6^. Urolithiasis itself is one of the risk factors for urinary tract infections. Therefore, patients with a history of urolithiasis surgery are more likely to develop recessive urinary tract infections even though their urine culture is negative, which may be an important reason for the higher probability of sepsis in patients with recurrent urolithiasis. Additionally, any clinical phenotype of UTIs has the potential to develop into severe urinary urosepsis^26^. Our study used UAS(F12/14) intraoperatively in all patients. UAS is a common way to maintain low intrapelvic pressure and reduce the risk of infectious complications^27,28^. UAS contributes to low irrigation pressure because it creates a pathway from the collection system to the outside of the body. Notably, these effects were most pronounced when large-diameter UAS was used^28^. Recent studies have shown that suctioning UAS^29^ and the novel flexible vacuum-assisted UAS^30^ are better at maintaining low intrapelvic pressure and reducing the incidence of postoperative infectious complications.

Previous studies have reported some independent risk factors related to postoperative infection complications of RIRS, such as the presence of urine nitrite^9^, positive UC^8,9^, large stone burden, and diabetes mellitus^31,32^. Therefore, for these high-risk groups, it is recommended to expand antibiotic prophylaxis and adjust the use of postoperative antibiotics. The AUA and EAU guidelines recommend single-dose antibiotics for patients receiving URS/RIRS, covering positive and negative cultures in all patients^2,33^. However, it is still based on limited evidence and rarely considers wide variations in individual patient characteristics. Therefore, it is necessary to stratify the risk factors for postoperative infectious complications in patients with negative UC before RIRS. A series of studies have shown that struvite composition^8,34^ and positive stone culture^11^ are also high-risk factors for postoperative sepsis. Still, postoperative sepsis generally occurs within 24 h after surgery, so these clinical indicators are of little significance for early predicting postoperative sepsis in patients. This study did not include these postoperative clinical indicators for analysis. Research by Cem Basatac shows that severe obesity is a risk factor for postoperative infectious complications after RIRS^35^. Our study did not find that obesity was statistically significant in the occurrence of sepsis after RIRS. The main reason for this controversy may be that Cem Basatac and his colleagues mainly focused on severely obese patients, all of whom had a BMI of more than 35 kg/m^2^. In the current study, however, these cases of severe obesity are relatively rare. And some small sample studies have shown that RIRS is a safe and effective surgical method for treating kidney stones in obese and overweight patients^36,37^. Therefore, the relationship between obesity and postoperative infectious complications of RIRS needs to be further studied in different populations.

In the current study, we constructed a nomogram based on these risk factors to predict the probability of postoperative infectious complications after RIRS in patients with negative urine cultures. The nomogram had great predictive power in the training and validation cohorts (AUC: 0.887 vs. 0.864). In addition, the calibration plot and decision curve analyses also performed well in the nomogram. Based on the optimal cut-off value of the training cohort, we found that the nomogram's corresponding cut-off value of the total score was 9.2. Patients with scores higher than 9.2 were at high risk for urosepsis after RIRS. Therefore, establishing our predictive model has important clinical values that could assist urologists in stratifying different risks of urosepsis at the very early phase for patients with negative preoperative UC According to this prediction model. Firstly, for patients with a low risk of urosepsis, prophylactic antibiotic use can be further shortened or even eliminated to avoid drug resistance caused by the overuse of antibiotics. Secondly, urologists should shorten the operation time appropriately. It is worth considering whether we should change the strategy of using prophylactic antibiotics in high-risk patients with negative preoperative UC, such as increasing the number of antibiotics used during the perioperative period. But there is a need to conduct a multicenter trial with a larger sample size to guide the best protocols to prevent RIRS infection in patients with negative UC; Thirdly, for high-risk patients, intraoperative lower-pressure irrigation is required, and we can use suctioning UAS or novel flexible vacuum-assisted UAS^30^ to maintain continuous low pressure in the renal pelvis. Fourthly, urologists must detect postoperative infection indicators and changes in basic vital signs such as body temperature, heart rate, and respiration in high-risk patients promptly to detect complications early and actively adopt anti-infection treatment strategies, such as using effective antibiotics to prevent the development of more serious complications, such as sepsis.

There were still certain limitations in the current study. First, this was a retrospective study in a single center with an inherent limitation; Second, This study aimed to establish and validate a preoperative nomogram for predicting postoperative urosepsis following RIRS in patients with a negative preoperative urine culture. Therefore, some postoperative clinical indicators, such as stone composition and stone culture, have not been included in this study; Third, relatively few urosepsis cases may lead to some instability in the results when multivariate logistic regression analysis is performed; Fourth, it is worth noting that the prediction model in our study was mainly for patients who received UAS during surgery, and may not be appropriate for patients who did not receive UAS. Considering these limitations of this study, further extensive prospective multicenter studies are needed to confirm the results.

## Conclusion

In this study, a nomogram combining preoperative independent risk factors can predict the probability of a postoperative urosepsis following retrograde intrarenal surgery in patients with a negative preoperative urine culture, which could help urologists take preventive measures in advance after surgery to avoid more serious complications of infection.

## Supplementary Information

Supplementary Information.

## Data Availability

Data will be made available on request. If required, the corresponding author can be contacted.
