# Supplementary material for: A nomogram for predicting postoperative urosepsis following retrograde intrarenal surgery in upper urinary calculi patients with negative preoperative urine culture
Source: Sci Rep. 2023 Feb 6;13:2123. doi: 10.1038/s41598-023-29352-y (PMC9902470; doi:10.1038/s41598-023-29352-y)
Supplement: Supplementary file 1 — Supplementary Information. [file 41598_2023_29352_MOESM1_ESM.pdf]

A nomogram for predicting postoperative urosepsis following retrograde intrarenal surgery in upper urinary calculi patients with negative preoperative urine culture

Authors: Miaomiao Yang [1], Yongchao Li [2], Fang Huang [2,*]

Supplementary Surgical procedure

After the patient was placed in the lithotomy position under general anesthesia, under the guidance of 4F ureteral catheters, 9.8-F semirigid ureteroscope (URS) (Karl Storz, Germany) was placed into the ureter to detect whether there was ureteral stenosis or deformity and a Zebra guidewire was inserted into the ureter through the URS. Then, the URS was removed and a ureteral access sheath (UAS, F12/14, 45cm for male, 35cm for female) was advanced into renal pelvis directed by the guidewire (If UAS implantation failed, double J tubes were implanted in the first stage, and the second procedure was performed two weeks later). Subsequently, the 9.9-F reusable digital FURS (URF-V; Olympus, Tokyo, Japan) was placed into the pelvis through the UAS. All patients were infused with gravity water bags (height 90-100 cm). Lithotripsy was performed using holmium:yt-trium-aluminum-garnet laser (Ho:YAG) with a 200-μm fiber at an energy level of 50–60 W and a frequency level of 15–24 Hz. The rubble fragments were recovered using a 2.4F zero-tip Nitinol stone basket (Cook Medical, Bloomington, IN, USA). After repeated examination of the collection system, it was confirmed that the stones were completely broken and removed. The operation ended with the placement of a double J stent in the ureter for drainage for 1 month.
